# Detection of Parkinson’s Disease Using Wrist Accelerometer Data and Passive Monitoring

**DOI:** 10.3390/s22239122

**Published:** 2022-11-24

**Authors:** Elham Rastegari, Hesham Ali, Vivien Marmelat

**Affiliations:** 1 Department of Business Intelligence and Analytics, Business College, Creighton University, Omaha, NE 68178, USA; 2 Department of Biomedical Informatics, College of Information Systems and Technology, University of Nebraska at Omaha, Omaha, NE 68182, USA; 3 Department of Biomechanics, College of Education, Health and Human Sciences, University of Nebraska at Omaha, Omaha, NE 68182, USA

**Keywords:** Parkinson’s disease, wearable accelerometer, early detection, passive monitoring

## Abstract

Parkinson’s disease is a neurodegenerative disorder impacting patients’ movement, causing a variety of movement abnormalities. It has been the focus of research studies for early detection based on wearable technologies. The benefit of wearable technologies in the domain rises by continuous monitoring of this population’s movement patterns over time. The ubiquity of wrist-worn accelerometry and the fact that the wrist is the most common and acceptable body location to wear the accelerometer for continuous monitoring suggests that wrist-worn accelerometers are the best choice for early detection of the disease and also tracking the severity of it over time. In this study, we use a dataset consisting of one-week wrist-worn accelerometry data collected from individuals with Parkinson’s disease and healthy elderlies for early detection of the disease. Two feature engineering methods, including epoch-based statistical feature engineering and the document-of-words method, were used. Using various machine learning classifiers, the impact of different windowing strategies, using the document-of-words method versus the statistical method, and the amount of data in terms of number of days were investigated. Based on our results, PD was detected with the highest average accuracy value (85% ± 15%) across 100 runs of SVM classifier using a set of features containing features from every and all windowing strategies. We also found that the document-of-words method significantly improves the classification performance compared to the statistical feature engineering model. Although the best performance of the classification task between PD and healthy elderlies was obtained using seven days of data collection, the results indicated that with three days of data collection, we can reach a classification performance that is not significantly different from a model built using seven days of data collection.

## 1. Introduction

Parkinson’s disease (PD) is a neurological disorder that occurs due to a decrease in the brain dopamine level, impacting patients’ movement. Symptoms of PD include movement abnormalities such as tremor, dysfunction of magnitude, and symmetry of arm swing, and gait characterized by shorter strides and slower movements compared to healthy individuals [1,2]. These symptoms have been shown to be detectable with a good amount of accuracy using body sensor networks and wearable technologies including wearable accelerometers [3,4]. The benefit of wearable technologies in the domain rises by continuous monitoring of this population’s movement patterns over time. Continuous monitoring seems to be a necessary step toward real-life applications of movement analysis in the context of the smart home and healthcare domain [5,6]. Monitoring patients in their own home over time provides opportunities for early detection of the disease and useful insights into the disease evolution, which in turn helps healthcare providers to assess the impact of various medications and interventions and come up with effective ways of slowing down the disease progression.

The wrist is the most common and acceptable body location for people to wear sensors on [7]. Advanced technologies such as smartwatches and activity trackers, which are being used by many people around the world, have provided us a platform to be able to monitor individuals’ wrist movement patterns continuously and remotely over time. Analyzing wrist movement patterns has been shown to be helpful in detection of freezing of gait events (FoG) and detecting tremor and dyskinesia in the PD population [7,8,9,10]. Early detection and tracking the evolution of the disease over time could be feasible using wrist accelerometers, as suggested by earlier studies [11,12,13]. In [12], authors reported a reduction in the amount and intensity of activity in patients with PD compared to healthy individuals using data from a sports watch. Wrist accelerometers have been shown to provide effective features for identifying PD patients from their healthy counterparts as well as the severity of the disease [13]. Early detection of PD has been shown to be feasible using wrist-worn accelerometry while passive monitoring the participants over a week [11].

The contributions of this study are listed as follows: (1) Early detection of PD using wrist accelerometer and passive monitoring, (2) using the document-of-word feature engineering approach to improve the performance over the traditional statistical feature engineering method, (3) identifying the best window size for segmentation of the wrist accelerometer data so that it increases the accuracy of the early detection of PD, and (4) identifying the optimal amount of monitoring time for the early detection of PD. Each of these goals that lead to our contributions in this paper are explained in the following paragraphs.

Passive monitoring of individuals over time using an easy-to-wear and comfortable sensor on the wrist could provide us with initial information on whether a person is likely to be in the early stages of a disease with movement disorder. This is the case for patients with PD, because PD is a progressive neurodegenerative disorder of the brain and central nervous system, affecting patients’ motor system, causing reduced movement, tremor, postural instability, and postural rigidity [14]. Fatigue, small shuffling steps, freezing of gait, and bradykinesia are some of the symptoms of PD [15]. If passive monitoring indicates any sign of the disease, then further investigation could be performed in the next step [11]. There are many research studies on detecting particular features of PD such as tremor, freezing of gait, and bradykinesia [3,16]. There is also a good amount of research studies on the early detection of PD in a control environment [17,18]. The only research study on the early detection of PD in an uncontrolled environment using wrist accelerometers is published by Williamson et al. [11], in which they used the Biobank dataset [19] collected from the participants’ dominant wrist. Our work will be among the very few studies using wrist accelerometers for the early detection of the disease. Plus, as suggested in [11], we should be able to rely on the early detection of PD using wearable technology regardless of which side of the body participants wear the wrist accelerometer (the most vs. less affected side and dominant vs. non-dominant side). While wearing an accelerometer on participants’ dominant wrist has been shown to provide good results [11], no study has reported on the early detection of PD by passive monitoring of participants’ non-dominant wrist. Moreover, using and combining symptom-based features with the other wrist movement features that are not based on the disease symptom could improve the sensitivity and specificity of the classification technique [11]. Therefore, our first contribution is the early detection of PD using wrist sensor data collected from the non-dominant wrist while combining symptom-based features with the other features.

The standard statistical feature engineering method has been the most common feature extraction method in this domain [20,21]. In this method, statistical features (e.g., vector magnitude) are extracted from signal subsequences of usually the same size. The document-of-words approach is a more recent approach in the domain of wearable signal processing. This approach has been shown to outperform the standard statistical approach in related domains such as real-life activity recognition, using data collected from accelerometers [22], and the early detection of PD, using accelerometers on participants’ left and right ankles [23]. Although the document-of-words feature engineering approach has been shown to provide good performance in PD detection using data collected from participants’ ankles, it was performed on a relatively small dataset with twenty participants. Moreover, it has never been used on accelerometers data collected from the participants’ wrist. Both approaches will be implemented and applied to our data. Then, the results will be compared to determine which approach has the highest performance.

Our third contribution is to test different window size values and identify the best one for data segmentation so that it leads to a classification model with the best performance for the early detection of PD. Choosing the right window size from which features are calculated has been shown to have a significant impact on detecting bradykinesia in patients with PD [3]. Choosing the right window size/sizes leads to a better identification of PD symptoms [3,4], which could in turn lead to a better model for the early detection of PD. Although the effect of feature window size has been studied for detecting PD symptoms, it has not been studied for the early detection of PD using wrist accelerometers, while it matters more in the context of passive monitoring in which there is no label for symptoms. In this study, we investigate the impact of different feature window sizes, when splitting the data, on our diagnostic models’ accuracy and efficiency.

The last contribution of this paper is to provide information on what could be the optimal amount of data needed for the early detection of PD when passive monitoring participants using a wrist accelerometer. The inertial sensors’ battery life depends on factors such as the number of sensors utilized, sampling frequency, and recording time, and even in the best-case scenario, when just accelerometer is utilized, there is a constraint on long-term data recording due to these devices’ limited battery lives [24]. Therefore, identifying the best possible amount of data needed for the early detection of PD without compromising the accuracy is a must-do task in this area. Compared to the time scale of hours, the time scale of days for monitoring PD patients has been found to better correlate with PD clinical metrics [25,26]. The total accuracy of a classification model for the classification of PD and healthy counterparts has been shown to improve gradually over a one full week time course [11]. The question would be when to stop collecting data without compromising the accuracy. In this study, we answer the question of how many days of passive data collection would provide us the best possible model for the early detection of patients with PD.

## 2. Related Works

Our main aim in this study is the early detection of PD in a free-living environment using wrist accelerometers. Many research studies have been conducted on PD clinical test automation using wearable technologies in the clinical setting [17,27,28,29]. A great deal of research focuses on automatic detection of PD symptoms, e.g., bradykinesia, tremor, freezing of gait, and rigidity in controlled environments such as clinics and biomechanics labs, using wearable devices [16,30,31]. To help PD patients recover from freezing of gait episodes, Gokul H. et al. proposed an embedded machine learning system from accelerometer data [32]. Work also has been performed for continuous monitoring of PD treatments such as levodopa [33]. Using sensors in a controlled environment, researchers have characterized wrist movement by variability measures in PD patients [34]. Using inertial sensors integrated in a smartphone combined with machine learning classification methods researchers could identify patients with PD [35]. Early detection of patients with PD is performed by researchers in controlled environments and mainly using gait features [36]. 

Our second aim is to investigate the impact of using two feature engineering methods on the early detection of PD: the statistical and document-of-words feature engineering methods. There are only a few research studies in which document-of-words feature engineering was performed. Kheirkhahan et al. [37] used the latter method in their work to identify activities of daily living and assess the energy expenditure per activity using wrist-worn accelerometers. The document-of-words feature engineering method was used by Rastegari et al. [23] to distinguish between PD and healthy elderlies using accelerometer data from participants’ ankles. Both studies showed a good outcome of the document-of-words feature engineering method. However, both used data collected in a controlled environment.

For related work, we consider studies in which a wrist sensor was used to collect data on individuals in free-living conditions, because the mean value of features calculated from data captured in a free-living environment has been shown to be different from that captured in a controlled environment [38,39].

Monitoring PD patients using wearable sensors and in a free-living environment has been attracting attention recently [40,41]. Monitoring in free-living conditions serves the following purposes for the PD population: early detection of the disease, identifying the disease severity level, and assessing the impact of a treatment or an intervention. In this study, our focus is passive monitoring of PD. While in active monitoring individuals are provided with a set of instructions on which tasks to perform and how to perform them, in passive monitoring individuals perform their activities of daily living without being instructed. Only three of the papers discussed in this section focus on the early detection of PD in free-living conditions using wearable technologies. 

In a research study by San Segundo et al., wrist accelerometry was used to detect tremor in PD both using annotated laboratory data and free-living data [41]. Their result indicated that tremor in PD can be detected with high performance using laboratory data and not using the free-living data, mainly because of the weak self-reported labels. However, the percentage of time detected with tremor was in good agreement with the self-reported labels. The result of this study cannot be compared with ours because it is not about the early detection of PD, and the performance could not be reported for the free-living environment. 

In a study conducted by Lipsmeier et al., individuals with PD and their healthy age-matched counterparts were monitored in free-living conditions using smartphones to investigate differences in features among participants [27]. Active (rest tremor, finger tapping, balance, and gait) and passive monitoring of individuals resulted in significant differences between all active and passive features from PD and controlled participants. Although they achieved 75% sensitivity and 81% specificity using the passive monitoring features, these results were not obtained using an independent test set.

Zhan et al. conducted a longitudinal study on PD subjects over a period of 6 months in which data were collected actively through five smartphone activities and used to develop a mobile PD score [17]. The score developed by them corelated well with several clinical tests including the Hoehn and Yahr scale and UPDRS (Unified PD Rating Scale) score. The result of this study cannot be compared to ours because they reported the coefficient of determination and not any accuracy, sensitivity, or specificity values.

The authors of the Parkinson@Home validation study [42] used accelerometers and gyrometers at five participants’ body locations, including the wrist, to distinguish between PD and healthy individuals. They reported accuracy and AUC values for the most and least affected wrist while the most affected wrist provided a better performance (AUC = 0.75 and accuracy = 57%). We use this study and its results to be compared with ours.

Habets J.G. conducted a study to monitor bradykinesia in patients with PD using a wrist accelerometer [3]. Their data were taken from the Parkinson@Home validation study, and they tried different windowing strategies to investigate the impact of those in the automatic identification of bradykinesia. They also compared group models (in which all subjects’ data were used) with individual models (in which each subject’s data were used separately and then the results averaged). Their results indicate that both group models and individual models perform at the same level (AUC = 0.70), which is the best performance obtained by increasing the window size to 300 s. This study can be used to compare our results with regards to different windowing strategies and with theirs.

The study of Williamson et al. on PD [11] is the most similar work to ours. The authors used the Biobank dataset [20], collected from the participants’ dominant wrist, and a Gaussian mixture model for the early detection of PD. In this study, analysis was performed based on two types of segments: gait segments and low-movement segments. They used gait dispersion features, low-movement correlation structure features, gait segments incidents, and the incidence of low-movement segments. They achieved good results with an area under the curve (AUC) of 0.85. The impact of different amounts of data in terms of number of days on model performance was also investigated in this study. Therefore, this study is a good comparison point with ours.

## 3. Materials and Methods

**Figure 1:**
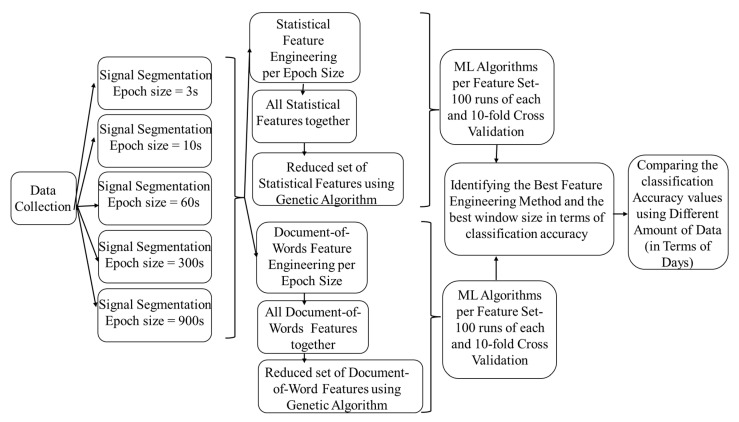
Steps to identify the best feature engineering method and the best windowing strategy/strategies.

**Figure 2:**
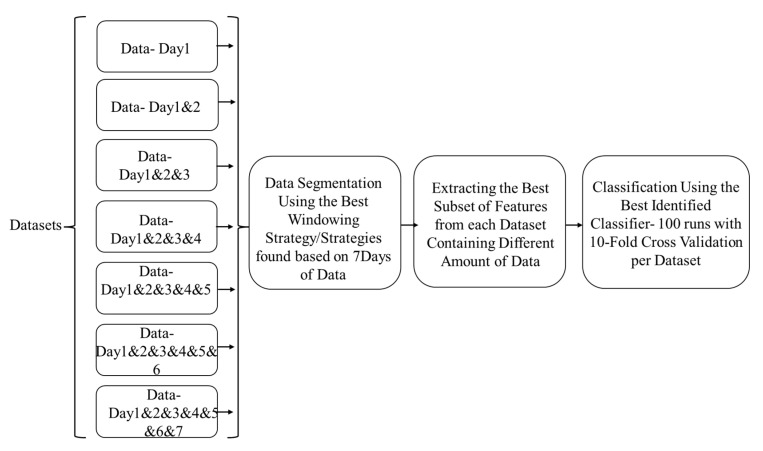
Steps to identify the optimal amount of data in terms of days.

The following five subsections of this section explain this study data collection’s protocol and methodology. Figure 1 shows the steps taken to identify the best feature engineering method and the best windowing strategy/strategies. Figure 2 indicates the steps taken to identify the optimal amount of data that need to be collected in terms of days.

### 3.1. Data Collection 

The data were collected from two groups of subjects: (1) healthy elderlies (64.2 ± 7 years and no movement deficiencies) and (2) individuals with various stages of PD (71 ± 6.2). PD patients reported no other illnesses impacting their gait and movement. The Hoehn and Yahr (H&Y) score used in our study is one of the commonly used measures of progression of PD and has been shown to correlate with motor decline happening in PD patients. The data were collected from participants while they had an Actigraph triaxial accelerometer on their non-dominant wrist for seven consecutive days. To be more specific, we used ActiGraph GT3X with ActiLife v6.13.3 Firmware v1.7.1 at 100 Hz. The sensor could be taken off while taking a shower or swimming. 

**Figure 3:**
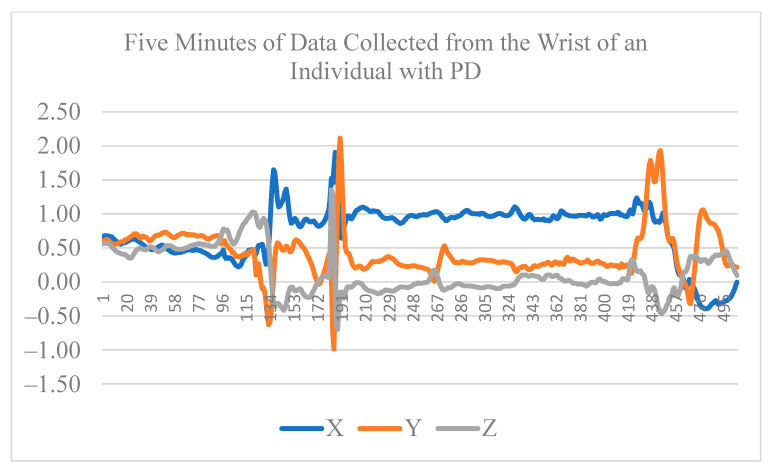
First five minutes of raw data collected from the wrist of an individual with PD.

**Figure 4:**
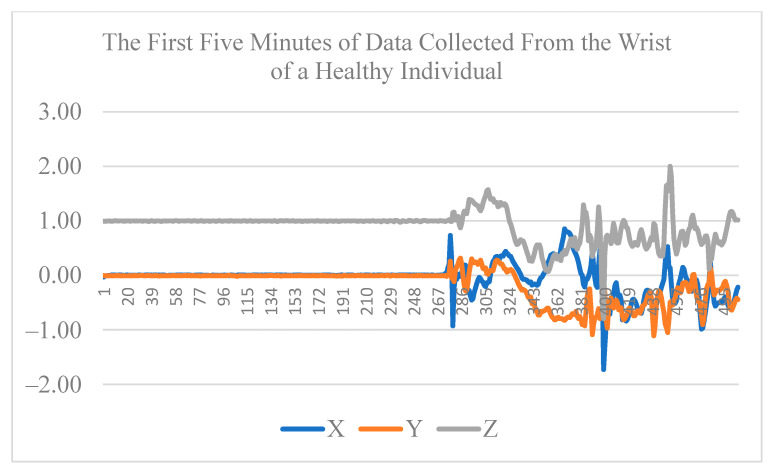
First five minutes of raw data collected from the wrist of a healthy individual.

The dataset includes three phases of data collection with 6–12 months between each two consecutive phases. The Actigraph device was recorded at 100 Hz with a range of ±8 g. In each phase, the H&R score and relevant clinical measures were recorded for each participant. The data from the first phase of the data collection is used for our analysis in this paper. Table 1 shows the participants’ characteristics. Figure 3 and Figure 4 show the first five minutes of raw data collected from the wrist of an individual with PD and a healthy individual, respectively. 

### 3.2. Signal Segmentation 

In this study, an epoch-based segmentation approach was used in which we converted each subject’s wrist movement signal into subsequences of a predefined length. Since a passive data collection was performed without labeling the data, each segment may represent both activities of daily living and symptoms of PD in the case of PD patients, which should be informative when it comes to distinguishing between the PD population and their healthy counterparts. Feature window size has been shown to significantly impact identifying symptoms of PD, including bradykinesia, resting tremor, and freezing of gait [3,7,16,43,44]. Therefore, different window sizes that are representing different signal granularity levels and could represent different symptoms of the disease were considered in our analysis. The classification task between PD and healthy elderlies was performed using the features obtained from each window size and also different combinations of different window size strategies. 

The window lengths considered in this study were as follows: (S ∈ {3 s, 10 s, 60 s, 300 s, and 900 s}). Each individual time series was split into smaller subsequences using a sliding window of S—second with overlapping windows of size $\frac{S}{2}$ seconds. These window lengths were chosen because they are likely to capture symptoms of PD. Three-seconds window length has been shown to be effective in capturing bradykinesia, tremor, and FOG using wrist accelerometer data [7,16], while increasing the window size to 300 s improves the average AUC of bradykinesia detection in PD patients [3]. In addition, using 5 min (300 s) epochs, researchers found that UPDRS III score is highly corelated with wrist accelerometer metrics [45]. A study of wrist accelerometer data for early detection of PD from a free-living setting used a sliding window of size 10 s and achieved a good AUC value [11]. A window length of 60 s has been commonly used in PD studies with the purpose of symptom detection [3,46]. We also consider a window length of 900 s (15 min) in our analysis, since investigating a window size larger than 300 s has been mentioned as a limitation of other PD studies due to their small sample size [3], which is not the case in our study. Moreover, bigger segments could be representative of activities of daily living.

### 3.3. Feature Extraction 

Using the epoch-based segmentation approach explained in Section 3.2, we used subsequences of data to extract features using two methods: standard statistical feature engineering and document-of-words method. To make the features independent from the accelerometer’s orientation, they were all calculated either over all three axes or the signal vector magnitude. All features were calculated over the subsequences of each windowing strategy. 

#### 3.3.1. Epoch-Based Statistical Features

To be able to compare the statistical feature engineering approach with the document-of-words method, a set of statistical features was used to create the epoch-based statistical features. Below is a list of features included in the analysis:

*Signal vector magnitude:* For the segments of each segmentation strategy, we calculated signal vector magnitude as √(X^2^ + Y^2^+ Z^2^) in which x, y, and z represent the accelerometers’ axes. This measure is dependent from the accelerometer’s orientation. This feature is used by researchers both for identifying PD symptoms [3] and early diagnosis of the disease [11] to identify a segment with hand movement versus a segment with low hand movement. Therefore, we assumed that this feature would help us in differentiating PD patients from their healthy counterparts.

*RMS of acceleration in the anterior posterior (AP), mediolateral (ML), and vertical (Ver) directions:* Root mean square (*RMS*) of acceleration is a time domain feature used in many studies focusing on PD symptom detection or early diagnosis of the disease [3,16,44]. Using the following equation (Equation (1)), we normalized the *RMS* values in each direction:

(1) $$RMSR_{dir}=\frac{RMS_{dir}}{\sqrt{{RMS_{AP}}^{2}+{RMS_{ML}}^{2}+{RMS_{Ver}}^{2}}}$$

The denominator is signal vector magnitude and *dir* represents each of the following directions: {*AP*, *ML*, *Ver*}.

*Movement dispersion feature:* this feature is introduced in [11] and only calculated on the segments identified as gait segments, not on the ones identified as low-movement segments. In this work, dispersion feature was calculated for all segments. Movement dispersion feature quantifies the average amount of acceleration variability within each segment. To calculate the dispersion feature vector for each segment, we used the method introduced by Williamson et al. [11]. First, we converted the acceleration values of each movement segment into their *z*-score values (zi(t) represents *z*-score of data point, *t*, in axis *i* within the nth segment). Then, we removed the outliers, defined as the points for which the standard deviation of either of the axes is greater than two. Then, using the valid points (non-outlier points) of each segment, dispersion in axis *i* can be calculated as follows in Equations (2) and (3):

(2) $$Dispersion_{i}\left(n\right)=\frac{\sum \;\left|\mathrm{Zi}\left(t1\right)-\mathrm{Zi}\left(t2\right)\right|}{S\left(n\right)}:\;\;\;t1,\;t2\in V(n)$$

(3) $$S\left(n\right)=\sum_{t_{1}t_{2}\in v_{n}}1\;$$

where *V*(*n*) represents a set of valid points within the nth segment, Zi(t) represents the *z*-score of data point t in axis I, and *S*(*n*) is the number of the pairs of valid points within the nth frame. Therefore, the vector of dispersion features for each segment looks like {Dx(*n*), Dy(*n*), Dz(*n*)}.

*Acceleration range:* This feature is reported among the features used for identifying tremor in PD patients [16,23]. To calculate this set of features, we took the difference between the minimum and maximum signal amplitude from three axes (ARAP, ARML, and ARVer).

The average of these features across all subsequences was calculated in the statistical feature engineering method and considered for classification model development. 

#### 3.3.2. Epoch-Based Document-of-Words Features

The document-of-words feature representation approach mainly includes three steps: data segmentation, vocabulary generation, and feature calculation, as described in [23]. Every subject’s data (i.e., 100 HZ accelerometer data for duration of one week per individual) are a time series sequence. Throughout the three main steps of this approach, every subject’s data were split into overlapping segments using a sliding window. Then, a clustering method grouped the similar segments into an appropriate number of clusters (explained in the following paragraphs), and similar segments within the same cluster were assigned the same word. Using this approach, each subject’s wrist movement time series was converted to a document of words. More details on this method can be found in the following paragraphs.

Data segmentation was performed using the method explained in Section 3.2. Once the segments or subsequences of data were generated, they needed to be represented using a feature descriptor set. This step was mainly needed for the next step (vocabulary generation), in which a clustering method identified the optimum number of clusters (words) based on the segments’ feature descriptor set. Signal vector magnitude has been used as a subsequence descriptor for assessing activities of daily living [37], while various combinations of different features are used to study the performance of the document-of-words model on identifying PD using ankle accelerometer data [23]. In this study, we used the best-identified feature set for the statistical features as our subsequences’ descriptor set (SDS). The number of total segments calculated for all subjects (considering that we have n number of subjects) per windowing strategy was calculated as follows (Equation (4)):

(4) $$N=\sum_{i=1-n}2\times \left(\frac{ti}{s}\right)-1$$

where *ti* denotes the duration of data collection and *s* is the window size. 

**Figure 5:**
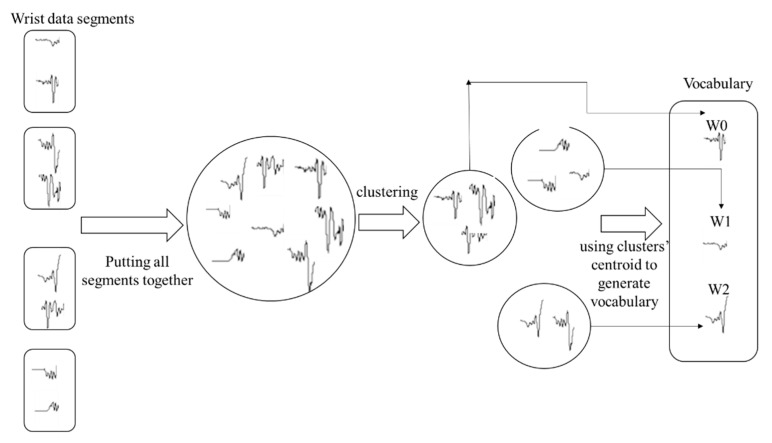
Epoch-based document-of-words feature engineering method—vocabulary generation phase.

In the second step, we generated a vocabulary set in which each subsequence was considered as a word. Since it is unlikely to find two identical subsequences with the same feature descriptors, we put all the segments obtained from all subjects together and grouped them using K-medoid clustering [23]. Therefore, segments that share similar characteristics are grouped together. To identify the number of clusters (k), we used the elbow method based on within-cluster sum of square (WCSS). The optimum number of clusters determines the number of words needed to represent all individuals’ wrist movement patterns. More details on how the elbow method using WCSS works can be found in [23]. Figure 5 depicts how the second step was performed. 

In the last step, every segment was assigned a word, which was assigned to its cluster centroid. Using these words, we could convert every subject’s wrist data into a document of words and construct a vector *D* = {*d0, d1, …, dk*} per subject’s data, in which di is a normalized value of word frequency based on the total number of words per individual (Equation (5)):

(5) $$tf\left(wi,D\right)=di=\frac{frequency\;of\;word\;wi\;in\;D}{total\;number\;of\;words\;in\;D}$$

### 3.4. Feature Selection, Classification Algorithms, and Validation

Feature selection is mainly performed in the analytics domain so that relevant features are selected for model development while irrelevant and redundant features are removed to reduce the model complexity and avoid overfitting. Since genetic algorithm (GA) has been shown to be one of the best algorithms for optimizing the classification of movement data in the healthcare domain, it was used for feature selection in our work. GA is a method inspired by natural selection to solve optimization problems by taking a population of individuals and trying to produce better approximations. The function to optimize here is the performance of our classification models. Each individual was considered to be a binary representation of features. A total of 40 random individuals were generated as the initial population. Our fitness function was defined as a combination of the average accuracy of all classifiers in this study combined with the number of features. The best fitness score is the one with the highest accuracy and the lowest number of features. We ran GA with 50 generations. In each generation, 20 individuals were selected for crossover, half of whom were the fittest and the other half random. This way, we avoided the issue of falling into a local optimum. Crossover was performed four times using random pairs, generating four feature sets. Using crossover, each feature set was generated by the features randomly selected from the parent feature sets. Then, each individual was given a 14% chance of being mutated, creating the new feature sets. More details on how genetic algorithm was applied to our feature set can be found in [47].

To create a model that distinguishes between healthy subjects and patients with PD, we applied different classifiers, including support vector machine (SVM with an RBF kernel), decision tree (DT), random forest (RF), K nearest neighbors (KNN), AdaBoost (AB), and naive Bayes (NB). These classifiers have been used in the gait analysis and document classification literature [5,23,48]. 

To evaluate each classification, we performed 100 runs of that classifier with 10-fold cross-validation, such that in each run of cross validation, nine folds (56 individuals’ data) were used for training and the remaining one fold (six subjects’ data) was considered as the validation set. Reported results are based on the average accuracy values and standard deviation around the average. This validation strategy is performed to make sure that the results are robust enough. 

### 3.5. Hypotheses Testing

#### 3.5.1. Comparing Statistical and Document-of-Words Feature Engineering Methods

To reach our second goal, in addition to the observations and results obtained from the classification models, we developed a hypothesis to compare the average performance of the two feature engineering methods. The following hypothesis was developed:

**H0:**  *mean_StatisticalFeatures_ ≥ mean_WordFeatures_*.

**H0a:**  *mean_StatisticalFeatures_ < mean_WordFeatures_*.

in which mean_StatisticalFeatures_ and mean_WordFeatures_ are the average accuracy values across all classifiers in the case of using statistical features and word features, respectively. The feature set used for this hypothesis test was generated using the best windowing strategy/the best combination of windowing strategies identified prior to this step.

Considering one set of features, 100 runs per classifier, and six classifiers, we must have 600 accuracy values per method. 

#### 3.5.2. Examining the Impact of Different Windowing Strategies on Classification Performance

To investigate the impact of window size on classification performance, we applied different classification methods using features calculated from subsequences of each windowing strategy (3 s, 10 s, 60 s, 300 s, and 900 s), different combinations of all features together, and a reduced feature set selected from the combination of all. The performance of the classifiers based on each single window strategy was considered as baseline. Considering 100 runs of each classifier and 10-fold cross-validation, firstly, all authors investigated the results by observation and identified windowing strategy/strategies that seemed to provide the best performance values. Then, we took the best ones and statistically checked for any significant difference between them and baseline windowing strategies. Here is an example of one hypothesis test developed:

**H0:**  *Performance_Combination_of_Aall_ ≤ Performance_3 Seconds_*.

**H0a:**  *Performance_Combination_of_All_ > Performance_3 Seconds_*.

in which Performance_Combination_of_All_ represents the average accuracy obtained using the features calculated from all data granularity levels, and Performance_3 Seconds_ represents the average accuracy of obtained using features calculated out of subsequences of 3 s length. Similar hypotheses were developed for the comparison of each pair of best performing window size/sizes and for the comparison of them with the baseline windowing strategies.

#### 3.5.3. Examining the Impact of the Data Size on Classification Performance

To identify the optimal number of days for data collection for early detection of PD, we selected the best windowing strategy/strategies and the best set of features (Section 3.5.2). Classification of PD against healthy elderlies was performed considering all possible number of days of data collection, consisting of one, two, three, four, five, six, and seven consecutive days. The best performing classification model and the best subset of features were used across all possible classification tasks based on different numbers of consecutive days. The classification task using each data size was performed 100 times with 10-fold cross-validation. Then, the accuracy values were taken for statistical analysis to compare the classification performance across different amounts of data. The developed hypothesis is as follows:

**H0:**  *performance_1 Day = performance_2 Days = performance_3 Days = performance_4 Days = performance_5 Days = performance_6 Day = performance_7 Day*.

**H0a:**  *not all the performance values from different amounts of data are the same*.

The null hypothesis states that the classification performance values are not significantly different across different numbers of days from which the data were collected. 

## 4. Results

### 4.1. Classification Results Using Statistical Features

Using the epoch-based statistical feature representation, the average value of each feature across all subsequences was calculated. First, all possible combinations of the statistical features were investigated by running the classifiers. The best results were obtained using SDS = {RMSX, RMSY, RMSZ, Vector Magnitude, RangeAP, RangeML, RangeVER, DX, DY, DZ}. Then, using this SDS, each classifier was run 100 times using features from various subsequence lengths. The best results were obtained using a reduced set of features containing at least one feature from each windowing strategy. 

**Figure 6:**
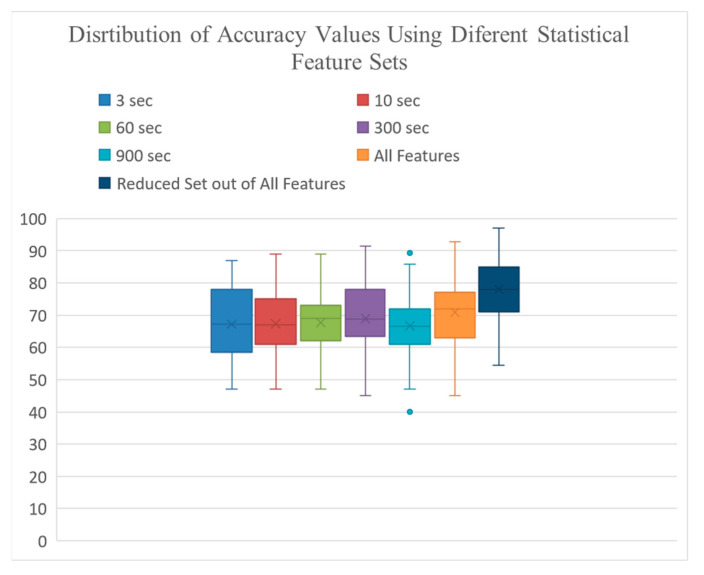
Distribution of accuracy values using various statistical feature sets.

Table 2 shows the average and standard error of accuracy values, and Figure 6 indicates the distribution of accuracy values obtained using features from different windowing strategies, a combination of features from all windowing strategies, and a reduced set of features. While we investigated all the combinations of different windowing strategies and their reduced subsets, we only listed the results for the baseline windowing strategies and two combinations with the highest performance.

As it appears, the best classification performance was obtained using a reduced set of features containing features from all windowing strategies. This set includes the following features: {DispersionAP_10 s, DispresionAP _60 s, RMSRAP-300, RMSRML-300, signal_vector_magnitude_300 s, signal_vector_magnitude_900 s, RMSRAP-3 s, ARML_3 s, ARAp_3 s, ARVer _3 s}.

### 4.2. Classification Results Using Document-of-Words Features 

To create the features using the document-of-words method, we used the same SDS introduced in the statistical feature method. Various values of subsequence lengths and various numbers of clusters were investigated. The optimum number of clusters/values of k were selected based on the elbow method for each and all windowing strategies. This number ranged between 4 and 32 for each window size. The value of k determines the number of words needed to represent all individuals. After investigating different values of k (k ∈ {4,8,12,16,20,24,28,32}), the optimum number of clusters for subsequences of 3 s, 10 s, 60 s, 300 s, and 900 s were found to be 22, 22, 20, 20, and 16, respectively. Therefore, each subject’s wrist movement signal could be represented as follows:

Pi-3 s ={D1, D2, D3, …, D22}

Pi-10 s = {D1, D2, D3, …, D22}

Pi-1 min = {D1, D2, D3, …, D20}

Pi-5 min = {D1, D2, D3, …, D20}

Pi-15 min = {D1, D2, D3, …, D16}

where Pi-3 s, Pi-10 s, Pi-1 min, Pi-5 min, and Pi-15 min represent the i_th_ person’s wrist data when the signal is split into subsequences of 3 s, 10 s, 1 min, 5 min, and 15 min, respectively. Combining all the words, each individual’s wrist data could be represented by a document of 100 words as follows:

Pi = {D1, D2, D3, …, D100}

**Figure 7:**
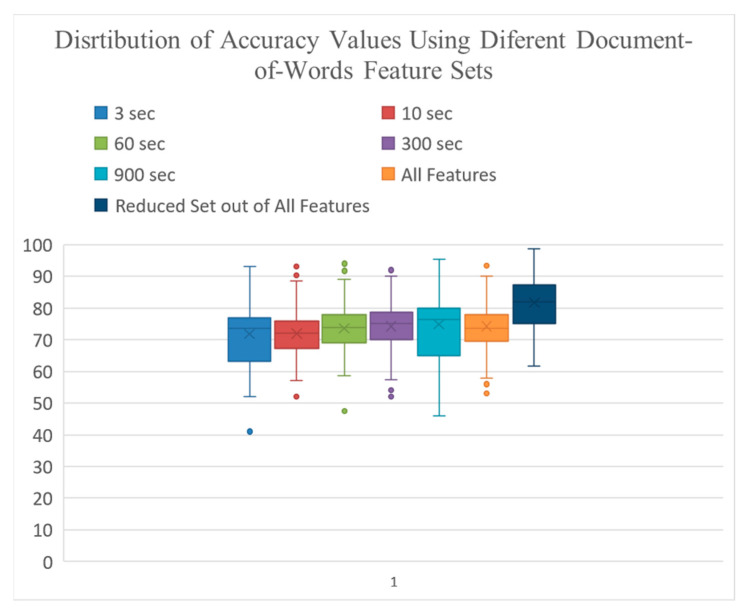
Distribution of accuracy values using various Document-of-Words feature sets.

Table 3 shows the average and standard error of accuracy values, and Figure 7 indicates the distribution of accuracy values obtained using the document-of-words features from different windowing strategies, a combination of features from all windowing strategies, and a reduced set of features.

### 4.3. Impact of Windowing Strategies 

For each hypothesis test, we first checked the normality, and since our data were normally distributed, we used ANOVA to compare the average accuracy values based on the different windowing strategies listed in Table 3. We only used the data from the document-of-words approach. Our result from a one-way ANOVA indicated that the means of the seven conditions were unequal—*F*(6, 593) = 4.07, *p* = 0.022. Tukey’s HSD test for multiple comparisons found that the mean accuracy value from the reduced feature set containing features from all granularity levels was significantly higher than that of all other features sets obtained from the other windowing strategies (*p* = 0.024, 95% confidence level), except for the one containing the combination of all features.

### 4.4. Comparing the Models Performance between Document-of-Words and Statistical Feature Engineering 

In general, if we look at the results in Table 2 and Table 3, we can see that the classification performance based on the document-of-words features is higher than that of the statistical features. To validate this observation statistically using a hypothesis test, we only selected the best classification results based on the windowing strategy/strategies out of each feature engineering method. Since the highest performance of each method was obtained using the reduced subset of features out of the combination of all window sizes, that feature set was used for our hypothesis test. 

The normality test was demonstrated that the distribution of accuracy values per group was normal. Therefore, a paired-sample t-test was performed. The test outcome indicates that the average accuracy value of the classification using word features is significantly greater that the average accuracy value of the classification using statistical features (*t* = 6.85, *df* = 4797.8, *p*-value = 1.27 × 10^−6^).

### 4.5. Optimum Number of Days of Data Collection

In order to find the optimum number of days of data collection for the identification of PD patients from the healthy elderlies, we identified the best classification performance, which was obtained from the reduced subset of twenty-words features. This subset of word features calculated over 7 days of data were used as our baseline so that we could compare the classification performance values based on different amounts of data collected over various numbers of days. This way, we could identify the optimum number of days for data collection that provides us with the same differentiating power as seven days of the data would. We only used the results of our SVM classifier with the RBF kernel for this section of the study.

Upon the result of the normality (*p* = 0.001) and homogeneity (constant variance) check, we could not use parametric test. Thus, the Kruskal–Wallis non-parametric test was selected and performed, which resulted in a chi-squared = 40.951, with *df* = 6, and *p*-value = 2.96 × 10^−7^. There is enough evidence that the classification performance considering different amounts of data (day-wise) is significantly different.

To perform a pairwise comparison, Dunn’s test with Bonferroni correction was performed. The results of Dunn’s test can be seen in Table 4. As can be seen in the table, considering one day of data provides significantly different results (lower accuracy) compared to other amounts of data, except for two days of data. Therefore, at least three days of data collection are needed.

## 5. Discussion

The contributions of this study are as follows: (1) Early detection of patients with PD using accelerometers worn on the most common and accepted body location, the wrist. Our results indicate that PD can be detected using wrist-worn accelerometry and passive monitoring of individuals with good accuracy values. (2) Application of the document-of-words feature engineering method, which is novel for PD detection using data collected from wrist sensors. Although this method was used for PD detection using data collected from participants’ ankles [23], the dataset was relatively small, and the data were not collected from wrist. Moreover, this is the first time that the impact of the document-of-words feature engineering method on early detection of patients with PD is statistically tested, showing that this feature engineering method significantly improves the classification performance compared to the traditional statistical feature engineering method. (3) Identifying the best windowing strategy/strategies for the early detection of PD using wrist accelerometers and passive monitoring of individuals. Although different windowing strategies have been tested to find the best one/ones for identifying the symptoms of the disease [3,7,11,16,45], there is no study in the literature that has investigated the impact of window size on the early detection of PD. Furthermore, the window sizes in this study are not picked randomly but are selected based on the best window sizes reported for the identification of symptoms of the disease [3,7,11,16,45]. (4) Identifying the optimum amount of data collection, in terms of the number of days, for the early detection of PD using wrist accelerometer data and passive monitoring. Considering continuous monitoring of individuals over time, which is a necessary step toward real-life applications of movement analysis in the context of smart homes and the healthcare domain [5,6], including a huge amount of data in analysis leads to a more computationally intensive process. If we can show that the same task can be completed using less amount of data and without compromising accuracy, it would help with saving memory and computational resources and speed up the early detection of PD. Therefore, an additional important finding of our study is that although collecting data over seven days improves people’s movement profiles and provide us with the best classification results, three days of data collection is not significantly different.

In this study, we used two feature engineering methods: epoch-based traditional statistical feature engineering method and the document-of-words approach. Our statistical feature set, which was also used as the subsequence descriptor set in the document-of-words approach, included the following set of ten features: {signal vector magnitude, RMSR_AP_, RMSR_ML_, RMSR_Ver_, Dispersion_AP_, Dispresion_ML_, Dispersion_Ver_, AR_Ap_, AR_ML_, AR_Ver_}. This set was selected as the best-performing set of statistical features. Various classification models were applied to our dataset using 10-fold cross-validation, and the results were reported per 100 runs of each classifier.

Based on our results, the document-of-words method significantly improves the classification performance compared to the statistical method. Interestingly, our results indicate that the highest performance of both the epoch-based statistical approach and the document-of-words method was obtained when we used a combination of features from all windowing strategies (3 s, 10 s, 60 s, 300 s, and 900 s). This set of features was a reduced feature set out of all possible features generated from each windowing strategy. The statistical set of features that contributed to the best classification results is as follows: {Dispersion_AP__10 s, Dispresion_AP_ _60 s, RMSR_AP_-300, RMSR_ML_-300, signal_vector_magnitude_300 s, signal_vector_magnitude_900 s, RMSR_AP_-3 s, AR_ML__3 s, AR_Ap__3 s, AR_Ver_ _3 s}. The reason why AR in all three directions using a window size of three is included in the selected feature set could be because of its importance in identifying tremor and bradykinesia [16]. The existence of RMSR_AP_ from 3 s window sizes could be explained using the same rationale. Dispersion features using a window size of 10 has been shown to contribute positively to the diagnosis of PD [11], and that could be why Dispersion_AP__10 s appeared as one of the important features in our reduced statistical feature set. Vector magnitude features from windows of sizes 300 and 900, and RMSR_AP_-300 and RMSR_ML_-300, can be also seen in the selected subset of features, which could be indicating the amount of hand movement over longer periods of time. Using the document-of-words features, the best performance was obtained using a reduced set of 20 words while the set had words generated from all windowing strategies. This indicates that considering data from each granularity level reveals some useful information about the populations in the study, and that contributes to a better performance of classification tasks.

This study is a step towards the early diagnosis of Parkinson’s disease using wrist-worn accelerometry in a real-life setting and would benefit patients and healthcare providers. The document-of-words feature engineering method eliminates the need for precise step/stride segmentation, which is a challenging task specifically for populations with a health condition.

It is not easy to compare our work and the results of this study with those from other studies because there has not been a great number of works that have attempted the early detection of PD using wrist-worn sensors and passive monitoring. Moreover, no study has reported on the impact of window size on PD diagnosis. Table 5 includes information on related works and specifies the similarities and differences between our work and theirs. As it may be noticed in the table, two studies that applied the document-of-words feature engineering method were both performed in controlled environments, one for the identifying of daily activities and the other one for PD diagnosis using accelerometry data from participants’ ankles [23,37]. By using participants’ ankle accelerometry, authors could achieve 80% accuracy [23] for the early diagnosis of PD, which is less than the average accuracy achieved in our study based on the document-of-words method and combining features from all windowing strategies. Lipsmeier analyzed data collected from PD patients and healthy age-matched individuals collected through passive monitoring and active scripted activates [27]. They reported 75% sensitivity and 81% specificity only using the turning speed feature but did not obtain these results using an independent test set. Moreover, they neither investigated the impact of different windowing strategies nor the impact of different amounts of data on models’ performance values. Evers et al.’s study focused on the diagnosis of PD using wrist-worn accelerometry and passive activity data collection [42]. By using data from the most affected wrist, they could achieve 0.75 AUC, which is less than the AUC value obtained in our analysis. The closest research study to our work is the one published by Williamson et al. [11], in which they used wrist-worn accelerometers and passive monitoring of the subjects. Since they reported AUC as the measure of performance in their study, we averaged AUC values obtained from 100 runs of the SVM classifier with 10-fold cross-validation and using the best subset of word features. We detected PD with average AUC = 86, which is very close to the results obtained by Williamson et al., who gained AUC = 0.85. This suggests that PD detection performance might be improved by considering low-movement and high-movement segments and their associated features, as reported in Williamson’s study [11]. Innovations of our work compared to Williamson et al.’s study [11] are as follows: we used the document-of-words feature engineering method, the impact of a different windowing strategy on classification models’ performance was investigated in our work, and we statistically identified the optimal amount of data that need to be collected for the early diagnosis of PD.

The methods and dataset used in this study have strengths and limitations. One of the strengths of the dataset is that it was passively collected from the most common and acceptable body location of individuals in a free-living condition. Moreover, our methodology does not require participants to wear the sensor on the most-affected wrist. Another strength of this work is that PD can be detected without explicit labeling of the data, e.g., symptoms detection and labeling. We did not have to label the data for activities of daily living and the symptoms of the disease. Furthermore, we had a balanced dataset in terms of the number of patients with PD and healthy elderlies. The other strength of our approach is that we had a set of features that was independent from sensor orientation.

Our dataset’s limitations are as follows: a limited number of participants and lack of additional information from each subject (e.g., if they presented any symptoms, and labeling the data or logs of their daily activities).

There are several opportunities for future work with the same dataset. Since we have three phases of data collection with 6–12 months in between, we should be able to test our results by combining participants from all three phases and see how that improves the results. Another possible task would be to select our test sets completely independently from the training set by selecting individuals for our test set from the other phase of the study. Another promising next step would be to conduct a longitudinal study and predict each individual’s health status in the third phase based on the first two phases of data. Another next step would be to develop models based on more sophisticated machine learning models to distinguish PD patients with different disease severities.

As mentioned in [11], wrist-worn accelerometry could be one modality of data collection that can be paired with other modalities for detecting PD and tracking disease severity. Combining wrist-worn accelerometry with other data sources, including sleep quality and speech analysis, could improve the power of PD detection algorithms and consequently improve this population’s quality of life.

## 6. Conclusions

This research study had four aims, including the early diagnosis of Parkinson’s disease in a free-living environment using wrist accelerometry, investigating the impact of two feature engineering methods on classification models’ performance, investigating the impact of different windowing strategies and their associated features on models’ performance, and investigating the impact of the amount of data on classification models’ performance. Our results indicate the feasibility of using data from wrist-worn accelerometry collected in free-living conditions for the early diagnosis of PD. The performance of our PD diagnosis models were improved using the document-of-words feature engineering method, and a subset of features including at least one feature from each windowing strategy was included. This indicates that including data from different granularity levels adds to our model performance. By comparing our models’ performance values using different amounts of data in terms of days, we noticed an increase in the model performance as the amount of data increased. However, our models’ performance values using three, four, five, and six days of data collection were not significantly different from that of seven days, suggesting the possibility of using three days of data collection for the diagnosis of PD without compromising the diagnosis model performance.

## Figures and Tables

**Table 1:** Subjects’ characteristics—phase 1.

	Healthy Elderlies	PD
Subjects	32	28
Gender (M/F)	10/22	20/5
Age	64.2 ± 7	71 ± 6.2
H&Y	-	1.73 ± 0.83

**Table 2:** Classification of PD vs. HE accuracy values—statistical feature engineering.

Window Size	Features	SVM	NB	DT	RF	KNN	AB
3 s	10	67.5 ± 18	68.4 ± 15	61.3 ± 18	67.1 ± 16	70.2 ± 18	62.2 ± 16
10 s	10	68.1 ± 16	67.1 ± 14	62.3 ± 15	68.2 ± 17	71.6 ± 18	63.4 ± 15
60 s	10	68.4 ± 17	69.4 ± 16	62.2 ± 19	69.2 ± 17	71 ± 18	62.3 ± 15
300 s	10	68.8 ± 16	68.8 ± 18	63.5 ± 18	68.1 ± 16	74.5 ± 17	66.2 ± 19
900 s	10	69.2 ± 16	61.9 ± 14	63.6 ± 16	69.4 ± 15	73.7 ± 16	60.8 ± 21
All	50	74.9 ± 18	74.2 ± 13	66.5 ± 16	74.8 ± 15	77.4 ± 14	61.8 ± 17
All	10	80.2 ± 14	81.1 ± 16	71.5 ± 17	79.2 ± 14	82.3 ± 14	71.1 ± 16

**Table 3:** Classification of PD vs. HE accuracy values using document-of-words feature engineering.

Window Size	Features	SVM	NB	DT	RF	KNN	AB
3 s	22	76.2 ± 17	75.2 ± 15	61.1 ± 19	76.6 ± 13	74.4 ± 15	69.8 ± 17
10 s	22	76.0 ± 17	74.1 ± 16	64.2 ± 17	75.3 ± 15	72.5 ± 15	70.5 ± 18
60 s	20	78.1 ± 16	77.2 ± 16	63.5 ± 16	77.7 ± 14	76.5 ± 18	69.0 ± 19
300 s	20	76.1 ± 16	75.3 ± 18	73.1 ± 19	76.2 ± 14	75.0 ± 17	70.1 ± 17
900 s	16	79.2 ± 15	79.1 ± 16	63.2 ± 17	78.0 ± 15	76.0 ± 16	74.3 ± 17
All	100	77.8 ± 17	75.6 ± 18	69.3 ± 16	78.2 ± 15	73.0 ± 17	72.0 ± 18
All-Reduced	20	88.5 ± 10	84.4 ± 14	74.8 ± 13	82.1 ± 14	84.7 ± 14	81.2 ± 15

**Table 4:** Dunn’s test pairwise comparison between the accuracy of classification using different amounts of data (day-wise). (Significant difference exist where “*” is observed).

Col-Mean Row Mean	Day1	Day2	Day3	Day4	Day5	Day6
Day2	−2.74 0.064	-	-	-	-	-
Day3	−3.96 0.0008 *	−1.22 1.000	-	-	-	-
Day4	−4.42 0.0001 *	−1.84 0.68	−0.69 1.000	-	-	-
Day5	−5.07 0.000 *	−2.70 0.07	−1.64 1.000	−0.96 1.000	-	-
Day6	−4.20 0.0003 *	−2.13 0.34	−1.20 1.000	−0.63 1.000	0.20 1.000	-
Day7	−4.32 0.0002 *	−2.25 0.25	−1.33 1.000	−0.75 1.000	0.095 1.000	−0.10 1.000

**Table 5:** Related Works with a Focus on Similarities and Differences between them and our Work.

Study	Free Living Condition	Sensor Location	Task	Features	Investigating Different Windowing Strategies	Investigating the Impact of Amount of Data on Models’ Performance	Perfomrance Measure	Performance Measure Value
This study	Yes- passive monitoring	Wrist	PD diagnosis	Document-of-Words (20 features from all and each window size)	Yes	Yes (collected 7 days of data)	Accuracy and AUC	0.88 and 0.86, respectively
[11]	Yes- passive monitoring	Wrist	PD diagnosis	{Dispersion, Correlation Structure Features}	No	Yes (collecrted 7 days of data)	AUC	0.85
[27]	Yes- passive and active monitoring	Smartphone	PD diagnosis	{Turning speed, sit-to-stand transitions per hour, activity ratio}	No	No (collected data over 6 months for PD and 45 days for Healty individuals	Specificity and sensitivity	81% and 75%, respectively, reported only for turning speed feature
[3]	Yes-active monitoring	Wrist	Bradykinesia detection	Statistical features, {maximum acceleration, coefficent of determination, root mean square, spectral power}	Yes	No (collected data for one hour pre medication and one hour post medication)	AUC	0.7
[37]	No	Wrist	Identification of activities of daily living	Document-of-words features	No	No (collected data from 4 visits performing activities of daily living as instructed)	F1-score	0.89
[23]	No	Ankle	PD diagnosis	Document-of-words features	Yes	No (collected data from one session walking 10 meters for four times	Accuracy, precision and recall	0.8, 0.7, and 0.9, respectively
[42]	Yes- passive monitoring	Wrist	PD diagnosis	{total power in 0.5–10 Hz, cadence, height of dominant peak, width of dominant peak}	No	No (collected data for one hour pre medication and one hour post medication)	AUC	0.75 for the most affected wrist and 0.49 for the least affected one

## Data Availability

Not applicable.
